# *HyperOptoNet*: a MATLAB-based toolbox for inter-brain neuronal synchrony analysis using fNIRS hyperscanning

**DOI:** 10.1117/1.NPh.10.2.025015

**Published:** 2023-06-13

**Authors:** Gihyoun Lee, Daeun Ro, Seyoung Shin, Yun-Hee Kim

**Affiliations:** a Sungkyunkwan University School of Medicine, Department of Physical and Rehabilitation Medicine, Suwon, Republic of Korea; b Sungkyunkwan University, Department of Health Sciences and Technology, SAIHST, Seoul, Republic of Korea; c CHA University School of Medicine, CHA Bundang Medical Center, Department of Rehabilitation Medicine, Seongnam, Republic of Korea; d Haeundae Sharing and Happiness Hospital, Pusan, Republic of Korea

**Keywords:** inter-brain synchrony, inter-brain neuronal network, cortical hemodynamic response, interpersonal brain network, functional near-infrared spectroscopy

## Abstract

**Significance:**

We developed a MATLAB-based toolbox for the analysis of inter-brain synchrony (IBS) and performed an experimental study to confirm its performance. To the best of our knowledge, this is the first toolbox for IBS based on functional near-infrared spectroscopy (fNIRS) hyperscanning data that visually shows the results on two three-dimensional (3D) head models.

**Aim:**

Research on IBS using fNIRS hyperscanning is a nascent but expanding field. Although various analysis toolboxes for fNIRS exist, none can show inter-brain neuronal synchrony on a 3D head model. In 2019 and 2020, we released two MATLAB toolboxes named *OptoNet* I and II, which have helped researchers to analyze functional brain networks using fNIRS. We developed a MATLAB-based toolbox named *HyperOptoNet* to overcome the limitation of the previous *OptoNet* series.

**Approach:**

The developed *HyperOptoNet* can easily analyze inter-brain cortical connectivity using fNIRS hyperscanning signals simultaneously measured from two people at the same time. The connectivity results can be easily recognized by representing inter-brain neuronal synchrony with colored lines that are visually expressed on two standard head models.

**Results:**

To evaluate the performance of the developed toolbox, we conducted an fNIRS hyperscanning study of 32 healthy adults. The fNIRS hyperscanning data were measured while the subjects performed traditional, paper-and-pencil-based, cognitive tasks or interactive, computer-assisted, cognitive tasks (ICT). The results visualized different inter-brain synchronization patterns according to the interactive nature of the given tasks; a more extensive inter-brain network was seen with the ICT.

**Conclusions:**

The developed toolbox has good performance of IBS analysis and helps even unskilled researchers to easily analyze fNIRS hyperscanning data.

## 1 Introduction

Traditionally, neuroscience studies focused on understanding the effects of stimulation on a single brain. However, this traditional “one-person” approach is now considered insufficient for investigating real-life social interactions, and neuroscientists are moving toward a “two-person neuroscience” approach.^1^ This approach stresses the need to understand how individuals influence each other by modulating a bidirectional information exchange.^2^ The rapidly growing interest in inter-brain neural synchrony (IBS) of multiple socially communicating brains, termed hyperscanning, is reflected in the number of review articles in the field using functional magnetic resonance imaging (fMRI), electroencephalography (EEG),^3^^–^^6^ and functional near-infrared spectroscopy (fNIRS).^7^^–^^9^ fMRI and EEG studies have shown that verbal and nonverbal communication induces interpersonal relationships among neural activities.^10^

fNIRS is a noninvasive optical imaging technique that uses near-infrared light to indirectly assess the metabolic activity of neurons in the outer layers of the cortex.^10^ fNIRS can measure changes in oxygenated hemoglobin (HbO) and deoxygenated hemoglobin (HbR) that correlate with the metabolic activity of neurons, similar to the blood oxygen level-dependent response obtained by fMRI.^11^^,^^12^ fNIRS is a promising method for investigating the relationship between interpersonal interactions in natural settings and their neural activities, and it has advantages of cost-effectiveness, low constraints on measurements, and relatively high tolerance to head and body motion.^13^^,^^14^ Recently researchers could better measure the inter-brain coupling associated with social interactions, and many studies about educational communication, a face-to-face game, improving cognitive performance, and body physiology between subjects have been reported using the fNIRS-based hyperscanning technology.^7^^–^^9^^,^^15^^,^^16^

Functional connectivity plays an important role in advanced cognitive processes and has drawn increasing attention from researchers over the past decades.^17^^–^^19^ Various methods exist for estimating functional connectivity, such as phase synchronization index,^20^ mutual information,^20^ partial directed coherence,^21^ frequency ratio,^22^ and mean phase coherence^23^ using multichannel neural signals including EEG, fMRI, and fNIRS.^24^ These functional connectivities have shown similar global functional connectivity patterns across studies, with some differences by cortical region, and have also shown agreement in quantifying the level of synchrony.^25^^,^^26^

There are many analysis tools for fNIRS, including hypergeometric optimization of motif enrichment (HOMER) from Harvard Medical School,^27^ near-infrared spectroscopy statistical parametric mapping (NIRS–SPM)^28^ from the Korea Advanced Institute of Science and Technology (KAIST), *OptoNet* series from Samsung Medical Center, and programs developed by each fNIRS system manufacturer. HOMER^29^ calculates individual hemodynamic responses using ordinary least-squares linear deconvolution, whereas NIRS–SPM^30^ applies the SPM method, which refers to construction and assessment of spatially extended statistical processes used to test hypotheses about functional imaging data.^28^^,^^31^ *OptoNet*,^32^ which was released by our research team in 2019,^31^ allows for easy and convenient cortical brain connectivity analysis based on three-dimensional (3D) finite element analysis.^33^ We also released *OptoNet* II in 2020;^34^ it can analyze according to functional region and automatic threshold settings to supplement the shortcomings of the previous version.^31^ However, there is no tool that can visually represent inter-brain neuronal synchrony from two fNIRS signals simultaneously measured through hyperscanning.

In this paper, we introduce *HyperOptoNet*, which is a MATLAB toolbox based on a Windows graphical user interface (GUI). Because *HyperOptoNet* includes most of the functions of the OptoNet series, it provides automatic thresholding and various functional connectivity estimating methods, such as correlation,^35^^,^^36^ coherence,^37^ frequency ratio,^22^ and phase locking value (PLV).^38^ Detailed descriptions and examples of *HyperOptoNet* are provided in the following sections.

We conducted an fNIRS hyperscanning study that separated 32 healthy subjects into two groups of 16, each of whom performed traditional, paper-and-pencil-based, cognitive tasks (TCT) or interactive, computer-assisted, cognitive tasks (ICT) to confirm the performance of the developed toolbox. The fNIRS hyperscanning data were measured while the subjects performed either TCT or ICT. We confirmed that the analyzed results could visualize different inter-brain synchronization patterns according to the interactive nature of the given tasks; the more extensive inter-brain network was seen with the ICT.

## 2 Methods and Materials

### 2.1 HyperOptoNet Procedures

**Fig. 1:**
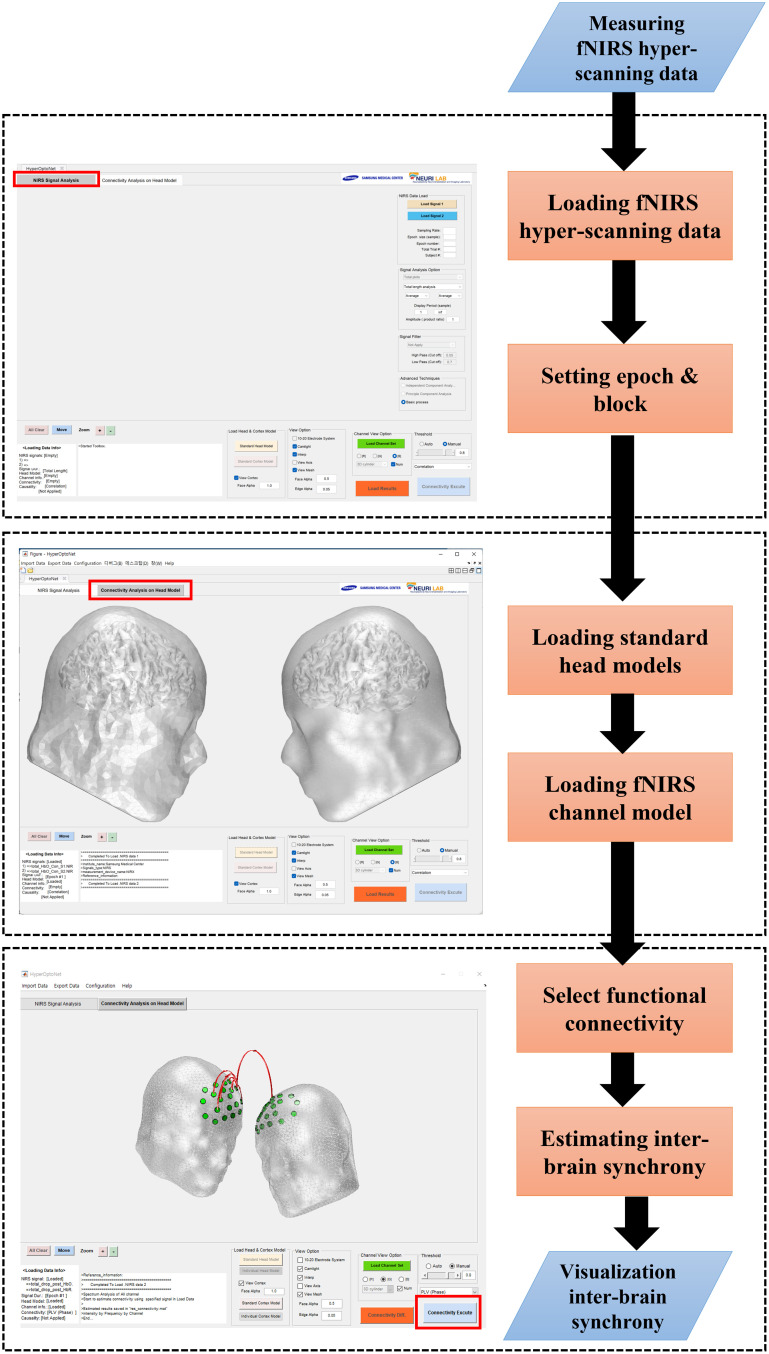
Sequential procedures of *HyperOptoNet.*

**Fig. 2:**
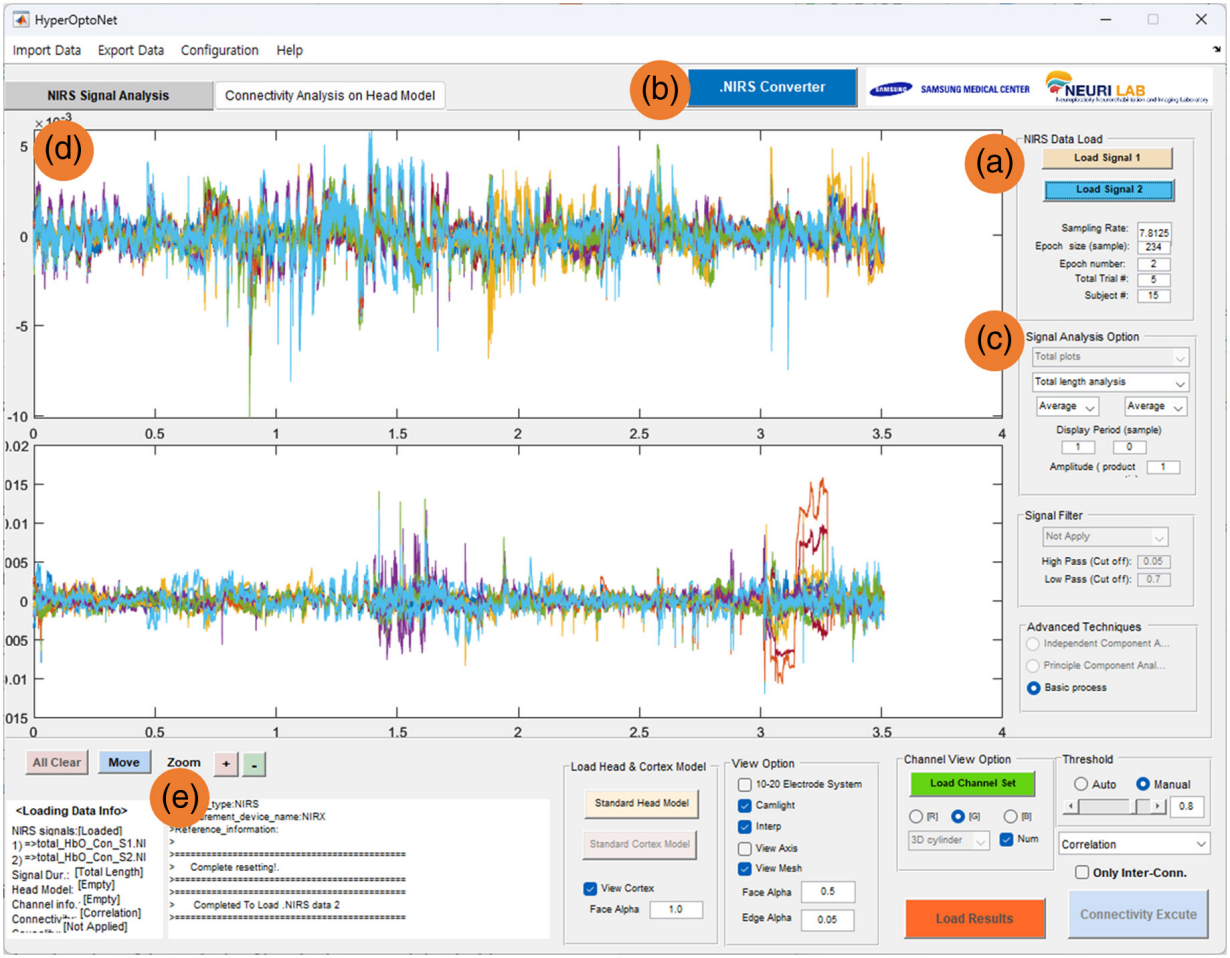
fNIRS hyperscanning data loading step: (a) “data load” section, (b) the button for loading.NIRS file converter, (c) “signal analysis” section, and (d) hyperscanning signal plotting panel, and (e) information panel.

The overall sequential procedures and GUI of *HyperOptoNet* are shown in Fig. 1. The procedures for analyzing inter-brain synchrony (IBS) can be roughly divided into the following steps: load fNIRS hyperscanning data, load the standard head and fNIRS channel model, and estimate IBS. *HyperOptoNet* is optimized for the latest version of MATLAB ver. R2022b (MathWorks, Natick, Massachusetts) on 64-bit Windows 10 and 11 (Microsoft Corporation, Redmond, Washington) installed in Intel i5 and i7 (Intel Corporation, Santa Clara, California) personal computer systems with GeForce (Nvidia, Santa Clara, California) graphics card series. Next, detailed descriptions of the *HyperOptoNet* procedures for each step are presented. The fNIRS hyperscanning data loading step is performed in the NIRS signal processing panel of *HyperOptoNet*. First, the measured fNIRS hyperscanning data are loaded in the “NIRS data load” section indicated in Fig. 2(a). the “load signal 1” and “load signal 2” buttons are used to sequentially load two fNIRS signals measured simultaneously through fNIRS hyperscanning. *HyperOptoNet* supports the.NIRS file format, which can be easily converted from the.mat file format of MATLAB. The *OptoNet* series provides an “.NIRS Converter” in Fig. 2(b), which can easily convert NIRx system’s fNIRS data and.snirf files to.NIRS files. The loaded fNIRS hyperscanning signals are plotted as shown in Fig. 2(d), and the plotting type, fNIRS epoch, experimental block, and subjects can be set in the “signal analysis section,” as shown in Fig. 2(c). When settings are changed, the fNIRS hyperscanning signals are refreshed in Fig. 2(d), and users do not need to execute this procedure more than once unless the fNIRS data are changed. Various system information and processing logs are displayed in Fig. 2(e).

**Fig. 3:**
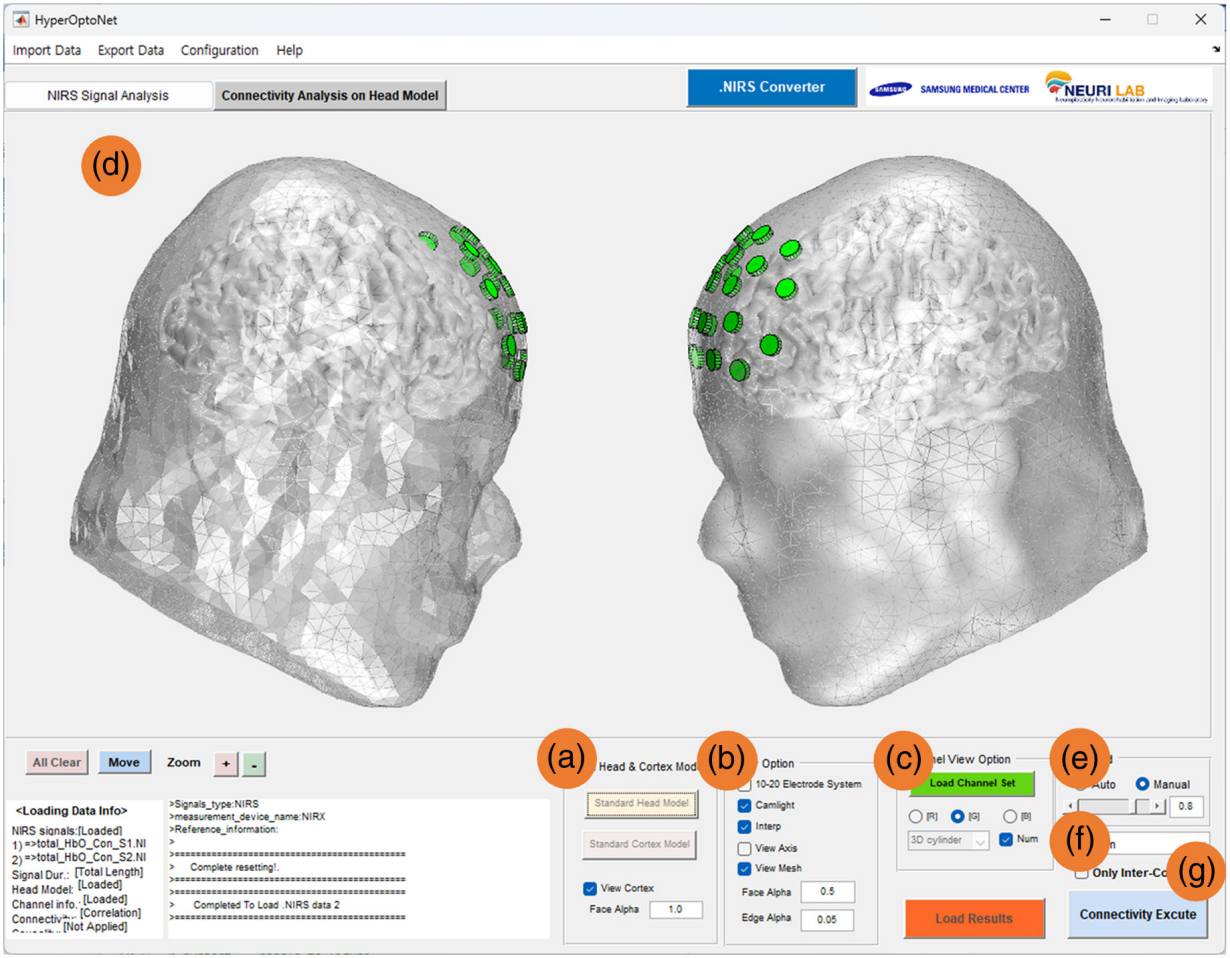
IBS step: (a) loading the head and cortex models, (b) viewing options of the head model, (c) section for loading the fNIRS channel set, (d) head model and synchrony display panel, (e) section for threshold selection, (f) selection of connectivity algorithms, and (g) the option for displaying only interbrain connectivity.

As shown in Fig. 3, the inter-brain neuronal synchrony estimating step is performed in the “connectivity analysis on head model” panel. Users can easily load standard head and cortex models in the “load head and cortex model” section by clicking the “standard head model” or “standard cortex model” buttons as shown in Fig. 3(a). There are many view options for the head model shown in Fig. 3(b), including viewing the 10-20 electrode system, camera light, axis, and/or mesh of the surface. Then, the channel set corresponding to the measured fNIRS channel needs to load using Fig. 3(c). The channel set is easy to create using the tool that is provided by the *OptoNet* series, and it is applied to both head models when only one channel set is imported. When the head model and the fNIRS channel appear as shown in Fig. 3(d), the tool is ready to estimate the inter-brain neuronal synchrony, and either automatic or manual thresholding can be selected, as shown in Fig. 3(e). If the manual threshold is selected, a specific value must be selected, but relatively fewer resources are needed. If the automatic threshold is selected, only the synchrony with a higher statistical significance is represented by autothresholding based on the surrogate test. Surrogate methods usually produce artificial data by randomizing the property to be tested while mimicking other properties (e.g., the spectra) of the original signal as much as possible.^24^ To choose significant synchrony, the toolbox follows a rank-order test.^39^ First, a residual probability α of false rejection is selected corresponding to a level of the significance [(1−α)×100%].^40^ Then, for a one-tailed test, which looks for small prediction errors, surrogate sequences (M=K/α−1) are generated, where K is a positive integer. For a two-tailed test, which can go both ways for time asymmetry, the surrogates (M=2K/α−1) are generated, resulting in a probability α that the data give either one of the K smallest or largest values.^40^ Using more surrogates increases the discrimination performance;^40^ 10,000 surrogate samples were used in *HyperOptoNet*. If synchrony has a higher surrogate than the level of the significance, it is represented as a color line on the head model. The algorithms used for the synchrony analysis can be selected from one of the following, as shown in Fig. 3(e): correlation, coherence, frequency ratio, and PLV. Correlation is one of the basic analysis algorithms for brain networks and analyzing statistical relationships, and it can have a good performance in analyzing the connectivity between two signals that have features in the time domain. Coherence is the statistical relationship of the power spectrum, and the frequency ratio is a ratio of the frequency of the pitches in two signals. Therefore, coherence and frequency ratio have good performances in analyzing the synchrony of brain signals with features in the frequency domain. Because PLV can analyze the intertrial variability of the phase difference between two signals, it has a good performance for brain signals, which have features in the phase and frequency domain, even if they have a time delay. Then the IBS can be estimated using the “connectivity execute” button. After estimation, only the synchrony greater than the threshold is represented, as shown in Fig. 3(d). The option to represent only IBS or with cortical connectivity can be on/off using the checkbox in Fig. 3(g). The result values of all estimated synchrony are saved in a work folder as a “res_connectivity.mat” file.

### 2.2 Participants

Thirty-two healthy adults (13 males and 19 females) between 65 and 84 years old (74.47±4.30 years) without a history of neurological disease participated. Subjects were randomly separated into two groups of 16 and assigned to the ICT or TCT group using a randomization program.^41^ The Institutional Review Board (IRB) of Samsung Medical Center, Seoul, South Korea, approved this study procedure (IRB No. 2020-08-147; clinical trial no. NCT04873843), and all participants provided signed informed consent before participating in the study.

### 2.3 Task Procedures

**Fig. 4:**
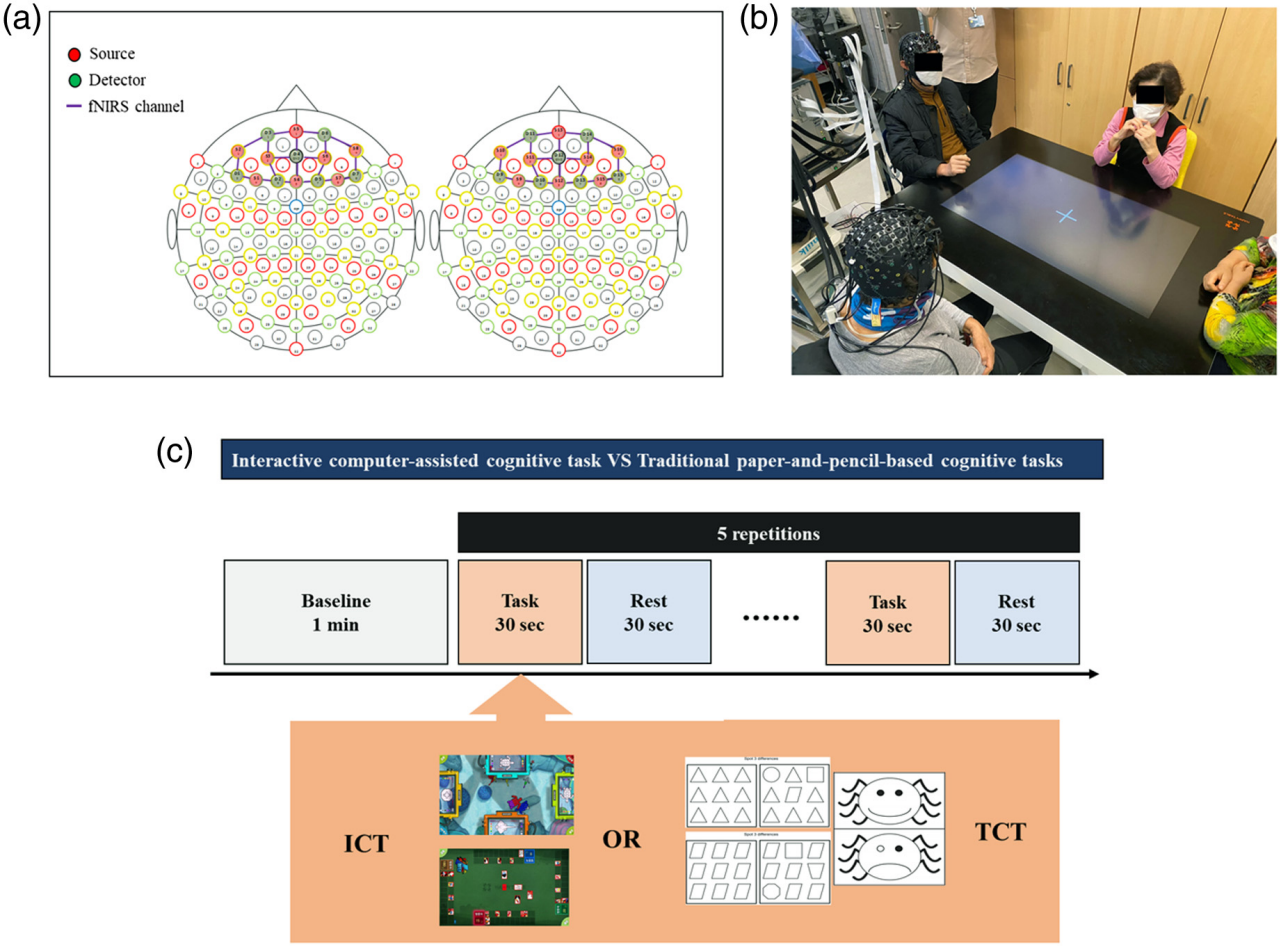
Experimental conditions: (a) the structure of the fNIRS channels, (b) measuring fNIRS hyperscanning data while subjects perform HAPPYTABLE®, and (c) experimental design and cognitive task paradigm.

The ICT group performed game-based interactive cognitive tasks using HAPPYTABLE^®^ (Spring Soft Co. Ltd., Seoul, South Korea), which is operable on a multitouch screen over a rectangular table, and four subjects can play together, as shown in Fig. 4(b). Each task was designed to include training in one or more cognitive areas involving basic information processing parameters and memory and requiring various types of decision making. The ICT group was provided 12 games that target the major cognitive domains of attention, visuospatial skills, memory, language, and executive function. Each game had multiple difficulty levels, and all participants played at the same difficulty level at the same time.

The TCI group consisted of cognitive stimulation programs widely used for older adults^42^ and included training in one or more cognitive areas organized from low to high levels in each intervention session. Nine cognitive tasks were chosen: mosaic, maze, finding the hidden picture, origami, spotting the difference, tangram, connecting the dots, coloring, and transcribing activities targeting the major cognitive domains.^43^^–^^45^ However, each subject performed the given tasks independently without interaction between participants.

### 2.4 fNIRS Hyperscanning Data Measurements

fNIRS hyperscanning data were measured by an fNIRS system (NIRScout^®^ 24-24, NIRx Medizintechnik GmbH, Berlin, Germany). This instrument has a light-emitting diode (LED) near-infrared light source with two wavelengths at 760 and 850 nm and a sampling rate of 7.8 Hz. The arrangement of the optode structure for hyperscanning fNIRS channels is shown in Fig. 4(a). To measure hyperscanning data, 16 near-infrared light sources and 14 detectors were divided into 8×7 each to produce 20 fNIRS channels in the prefrontal area for two subjects each. The fNIRS optodes were divided into two sides based on the location of the fNIRS device and attached to the two subjects. Hyperscanning data from both people were simultaneously measured during the cognitive task, as in Fig. 4(b). The task paradigm is shown in Fig. 4(c), starting with 1 min in a closed-eye state to obtain the baseline of the fNIRS signal. Subjects then performed the cognitive tasks for 30 s, rested for 30 s, and repeated these blocks five times. Thirty-two subjects visited twice, produced 64 hyperscanning data points, and were analyzed with 64 fNIRS data samples.

### 2.5 Inter-Brain Neuronal Synchrony Analysis

The oxy-hemoglobin (HbO) signal was estimated from optical density, which was measured by the fNIRS system using the NIRS-SPM toolbox^28^ in MATLAB. A band-pass filter (0.01 to 2 Hz) was used as the preprocessing of the raw HbO signals to maintain the low-frequency oscillation, respiration, and cardiac information in fNIRS signals.^46^^,^^47^ Inter-brain neuronal synchrony was estimated using the PLV. It can detect synchrony in a precise frequency range between two recording sites, uses responses to a repeated stimulus, and searches for latencies at which the phase difference between the signals varies minimally across trials.^38^ The PLV measures the intertrial variability of this phase difference: if the phase difference varies minimally across trials, PLV is close to 1; otherwise, it is close to 0.^38^ Therefore, PLV is suitable for analyzing the synchrony of fNIRS signals.

## 3 Experimental Results

**Fig. 5:**
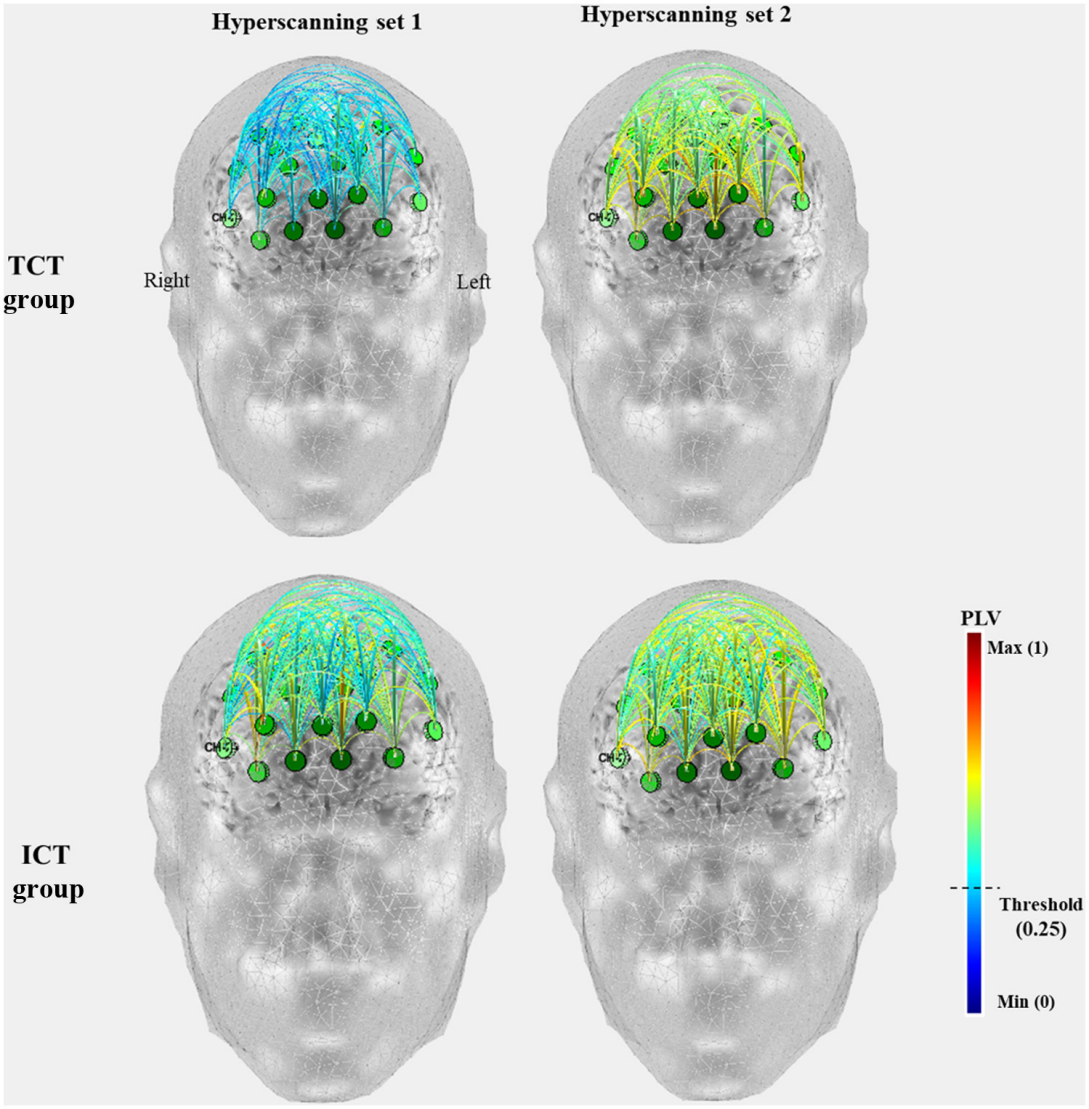
Results of cortical connectivity using *OptoNet* II.

The functional connectivity results obtained using the *OptoNet* are shown in Fig. 5 and were obtained based on fNIRS channels with manual thresholding (PLV>0.25). As can be seen in the results in Fig. 5, the ICT group had higher functional connectivity between fNIRS channels than the TCT group. However, through the results of functional connectivity, IBS between subject groups 1 and 2 could not be analyzed using the *OptoNet*.

**Fig. 6:**
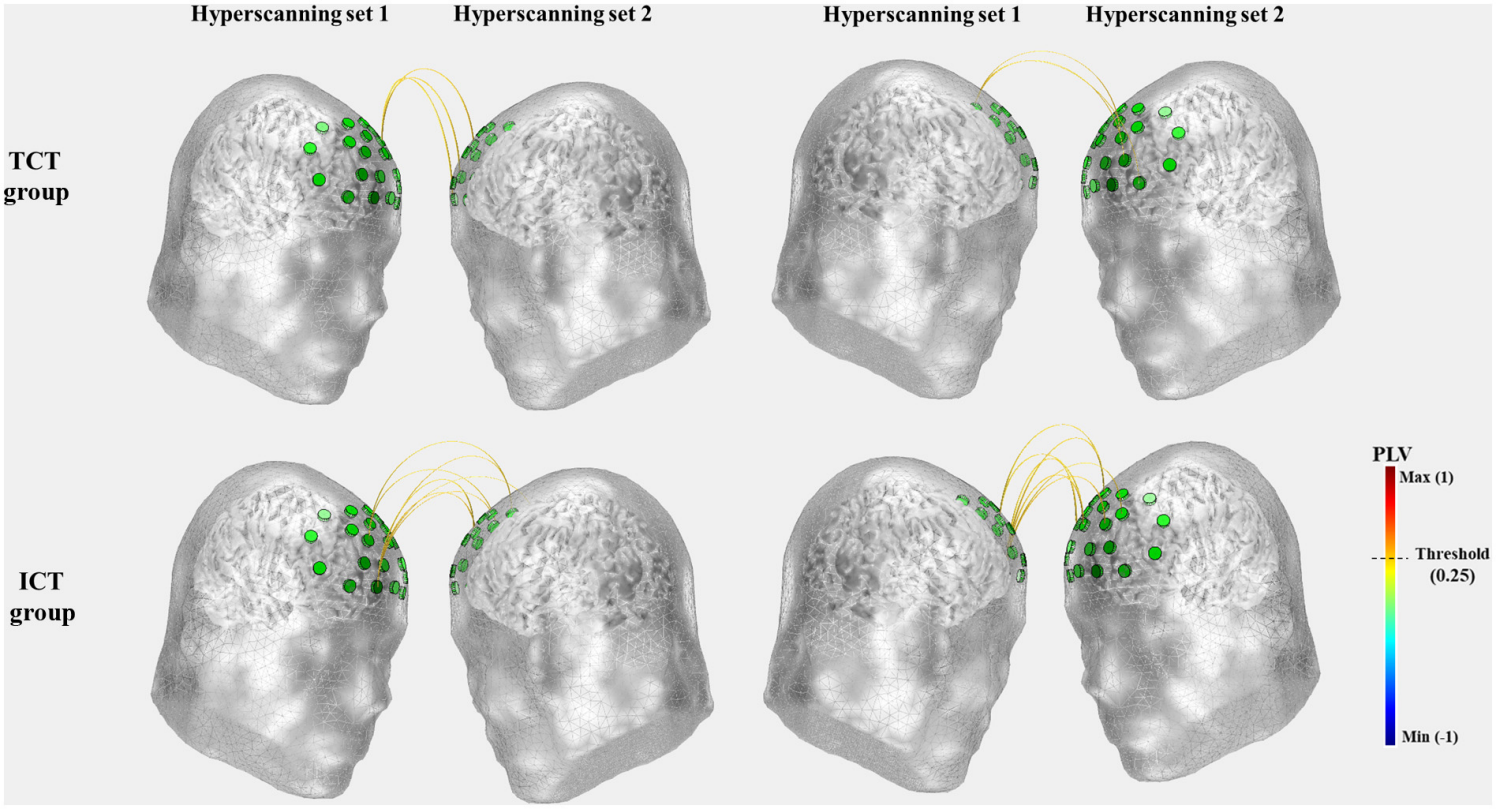
Results of inter-brain neuronal synchrony using *HyperOptoNet*.

To analyze IBS, the signal length settings for analysis in “signal analysis” in Figs. 5 and 6 were set as “total length.” In this setting, the connectivity of the whole fNIRS signals was estimated without signal averaging between blocks and trials. The IBS of the two subjects who performed the task at the same time was estimated, and then the averages of the IBS for each trial were calculated and are represented in Fig. 6. The results of the ICT group show more synchrony connections that have a PLV value >0.25 than in the TCT group (ICT group: 7, TCT group: 3). The average value of inter-brain synchronies in the ICT group is higher than the TCT group (ICT: 0.1235±0.0529, TCT: 0.1075±0.0541). In the TCT group, only the synchrony connections between the dorsolateral prefrontal cortex (DLPFC) are shown, whereas in the ICT group, the synchrony connections are shown in various areas of the prefrontal cortex.

## 4 Discussion

We previously released two MATLAB-based toolboxes, named *OptoNet* I and II, to analyze functional cortical connectivity for fNIRS. It is easy to use to analyze functional cortical connections using hyperscanning fNIRS signals without any anatomical information. An emerging body of hyperscanning fNIRS research shows inter-brain neural synchrony during different forms of social interaction.^16^ In this study, we developed a new toolbox called *HyperOptoNet* that, unlike the *OptoNet* series, can analyze inter-brain neuronal synchrony to analyze IBS. The interest in inter-brain neural synchronization by measuring multiple socially communicating brains has been rapidly growing in the fNIRS field. The research showed inter-brain neural synchrony during different forms of naturalistic social interactions, including body movement coordination and imitation,^48^ side-by-side playing of a computer-based cooperation game,^7^ educational communication,^8^ improving cognitive performance,^9^ body physiology,^15^ and face-to-face communication.^49^ To confirm the performance of the developed toolbox, we measured fNIRS hyperscanning data in two groups of 16 subjects performing ICT or TCT. The analyzed results were visually shown in two standard head models, and there was different inter-brain neuronal synchrony according to the interactive nature of the given tasks. In the previous fNIRS hyperscanning study, strong IBS was observed in the middle frontal gyrus and superior frontal gyrus for participants playing both a cooperative and an obstructive Jenga game,^48^ and parent–child dyads exhibited IBS in the DLPFC and frontopolar cortex during a cooperative task.^50^ In this study, IBS was observed between DLPFCs in the TCT group (Fig. 6), which seems to be the reason that DLPFC has various roles in cognitive control.^50^ Furthermore, the results of the ICT group showed more IBS than in the TCT group and showed IBS in various areas such as the DLPFC as well as the dorsomedial prefrontal cortex (DMPFC). This result is similar to those of previous studies, in which IBS appeared only for the cooperative condition, within the DMPFC.^49^

In this study, we developed and released a new MATLAB-based toolbox for the analysis of IBS and performed an experimental study to confirm its performance. To the best of our knowledge, this is the first toolbox that can analyze inter-brain neuronal synchrony based on fNIRS hyperscanning data and visually show the results on two 3-D head models. We hope that our toolbox can contribute to the popularization of IBS analyses in the field of fNIRS research.

## 5 Conclusions

In this work, we developed a freely downloadable MATLAB-based toolbox, named *HyperOptoNet*,^51^ to analyze inter-brain neuronal synchrony. Constructive comments and questions are always welcome through our e-mail. In this study, although a significant difference in inter-brain neural synchrony for each group was not found, the validity of the developed toolbox could be verified, and this toolbox does not provide individual channel or size settings for each of the two head models yet. For future studies, OptoNet series including *OptoNet* I and II and *HyperOptoNet* will be continuously updated in both the individual settings and with additional methods for the analysis of functional connectivity and multiple comparison correction. We plan to enable this toolbox not only for functional network analysis but also for brain activity monitoring during transcranial electrical stimulation, so it can contribute to expanding fNIRS research fields.
